# Complete plastome sequence of *Rhodoleia championii* Hook. f. (Hamamelidaceae)

**DOI:** 10.1080/23802359.2019.1674702

**Published:** 2019-10-09

**Authors:** Hai-Li Li, Xia-Lan Cheng, Yan Chen, Fei-Li Tan

**Affiliations:** Life Science and Technology School, Lingnan Normal University, Zhanjiang, Guangdong, China

**Keywords:** *Rhodoleia championii*, Chloroplast genome, Hamamelidaceae

## Abstract

The complete chloroplast genome of *Rhodoleia championii* (Hamamelidaceae) was firstly reported in this study. The complete chloroplast genome of R. championii is 159,115 bp in length with a typical quadripartite structure, consisting of a large single-copy region (LSC, 88,123 bp), a single-copy region (SSC, 18,131 bp) and a pair of inverted repeats (IRs, 26,420 bp). There are 114 genes annotated, including 80 unique protein-coding genes, 4 unique ribosomal RNA genes, and 30 transfer RNA genes. According to the phylogenetic tree of *R. championii* and the other 9 species, *R. championii* was closely related to *Chunia bucklandioides*.

*Rhodoleia championii* Hook. f. belongs to the family Hamamelidaceae. It is a tree of evergreen broad-leaved forests occurring at an altitudinal range of 700–1000 m in Guangdong Province and the neighbouring provinces Hong Kong, Guizhou, and Hainan (Yang et al. 1983).

As chloroplast carry maternal genes, it is important in phylogeny reconstruction. However, there have been no published plastome sequences for *R. championii* chloroplast to date. The genetic and genomic information is urgently needed to promote its systematics research and develop molecular markers to improve its aesthetic trait. Here we report and characterize the complete plastid genome sequence of *R. championii* (GenBank accession number: MK834325) in an effort to provide genomic resources useful for promoting its conservation and new species cultivation.

In this study, the fresh leaves of *R. championii* were collected from the biological garden of Lingnan Normal University (110°20′53″E, 21°16′04″N). Voucher specimens (LNH180530010) were deposited in the Herbarium of Lingnan Normal University, Zhanjiang, China. The experiment procedure was as reported in Liu et al. (2016). Total DNA of the *R. championii* was sequenced with second-generation sequencing technology (Illumina HiSeq 2000, San Diego, CA). The chloroplast genome sequence reads were assembled with bioinformatic pipeline including SOAP2 software (Li et al. 2009) and several runs of manual corrections of sequence reads. Genes encoded by this genome were annotated by importing the FASTA format sequence to the DOGMA (Wyman et al. 2004) and recorrected by manual. The results showed that plastome of *R. championii* had a total length 159,115 bp with the typical quadripartite structure of angiosperms, containing two inverted repeats (IRs) of 26,420 bp each, a large single-copy (LSC) region of 88,123 bp, and a small single-copy (SSC) region of 18,131 bp. The plastome contains 114 genes, consisting of 80 unique protein-coding genes, 30 unique tRNA genes, and 4 unique rRNA genes. The overall A/T content in the plastome of *R. championii* is 62.30%, and the corresponding values of the LSC, SSC, and IR region were 64.20, 67.70, and 57.10%, respectively.

We used RAxML (Stamatakis 2006) with 1000 bootstraps under the GTRGAMMAI substitution model to reconstruct a maximum-likelihood (ML) phylogeny of 9 published complete plastomes of Saxifragales, using *Paeonia lactiflora* and *Paeonia decomposita* (Paeoniaceae) as outgroups. According to the phylogenetic topologies, *R. championii* was closely related to *Chunia bucklandioides.* Most nodes in the plastome ML trees were strongly supported (Figure 1). The complete plastome sequence of *R. championii* will provide a useful resource for the conservation genetics of this species, as well as for the phylogenetic studies of Saxifragales.

**Figure 1.**
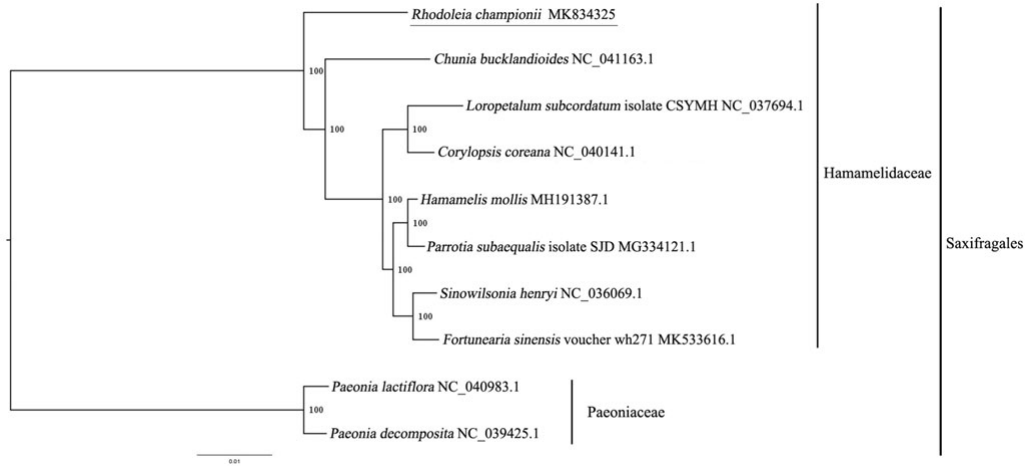
Maximum-likelihood phylogenetic tree based on 10 complete chloroplast genomes. Accession number: *Rhodoleia championii* (this study); *Chunia bucklandioides* MG644608.1; *Loropetalum subcordatum* isolate CSYMH NC_037694.1; *Corylopsis coreana* NC_040141.1; *Hamamelis mollis* MH191387.1; *Parrotia subaequalis* isolate SJD MG334121.1; *Sinowilsonia henryi* MF49744.1; *Fortunearia sinensis* voucher wh271 MK533616.1; *Paeonia lactiflora* NC_040983.1; *Paeonia decomposita* NC_039425.1. The number on each node indicates the bootstrap value.

## References

[CIT0001] Li RQ, Yu C, Li Y, Lam TW, Yiu SM, Kristiansen K, Wang J 2009 SOAP2: an improved ultrafast tool for short read alignment. Bioinformatics. 25:1966–1967. 19497933 10.1093/bioinformatics/btp336

[CIT0002] Liu J, Niu YF, Liu SH, Ni SB 2016 The complete pineapple (*Ananas comosus*; Bromeliaceae) varieties F153 chloroplast genome sequence. Mitochondrial DNA Part B. 1:390–391. 10.1080/23802359.2016.1174084 PMC7799915 33473493

[CIT0003] Stamatakis A 2006 RAxML-VI-HPC maximum likelihood-based phylogenetic analyses with thousands of taxa and mixed models. Bioinformatics. 22:2688–2690. 16928733 10.1093/bioinformatics/btl446

[CIT0004] Wyman SK, Jansen RK, Boore JL 2004 Automatic annotation of organellar genomes with DOGMA. Bioinformatics. 20:3252–3255. 15180927 10.1093/bioinformatics/bth352

[CIT0005] Yang YY, Zeng TS, Xu YQ 1983 On the main forest-types and the structure characters of the communities in Nan Kun Mountain. J South Chin Agri Univ. 4:11–20.

